# The Immediate Treatment of Puerperal Sepsis

**Published:** 1909-01-30

**Authors:** 


### Obstetrics.

### THE IMMEDIATE TREATMENT OF PUERPERAL SEPSIS.

### III.—Acute Puerperal Endometritis.

Although the majority of cases of puerperal sepsis are sapræmic in type, there are a fair proportion of infections which are not sapræmic and yet are not true septicæmias. These cases are essentially infections of the uterus at the placental site, and the lesion may be termed acute puerperal endometritis. Such conditions without the bacteriologist's aid are differentiated from true septicæmias with great difficulty; very often the diagnosis has to be made by a process of exclusion.

#### Symptoms.

Patients suffering from this condition are very ill, much more so than in the sapræmic type, and have temperatures reaching 104° or 105° F., with remissions to 99° or 100°; but they do not always have rigors. They may have one rigor at first, but usually no more. In true septicæmia more than one rigor is the rule, and the temperature keeps high without marked remissions.

The patient is generally markedly anæmic, may vomit or have diarrhœa, and always has a quick, soft, compressible pulse. Locally the uterus is found to be much higher above the pubes than it should be, and is often tender: tenderness, however, is not nearly so marked as is local pain in the sapræmic cases. The lochia are normal in colour, or redder than normal, never suppressed; they show no evidence of decomposition. Suppression of the lochia only occurs in the very worst types of acute septicæmia. The interior of the uterus on exploration in the routine manner already described reveals no retained material, or very small shreds; and the contents of the uterus are not foul. This latter fact at once serves to distinguish these from the true sapræmic cases. In the vast majority of cases of this kind cultures made from the interior of the uterus reveal a streptococcus infection, sometimes quite pure, but occasionally in combination with the staphylococcus aureus and the bacillus coli communis. These are the cases in which the immediate bacteriological investigation at the first examination may prove of the greatest value in indicating the proper line of treatment.

#### Local Treatment.

Great discussions have taken place as to what is best to be done to the uterus itself in such cases. The bulk of authoritative opinion is against curettage, the chief argument being that the removal of the lining of the uterus lays bare flesh areas for the organisms to attack. The lining of the uterus is crowded with leucocytes which form a barrier against a generalised infection, and the very fact that the infection is localised to the uterus proves the efficacy of this leucocytic army. Moreover, thorough curettage of the large uncontracted flabby uterus is impossible, to say nothing of the danger of perforation if the operation be attempted. Further, it is impossible to remove or destroy all the organisms in the uterus by curettage or strong antiseptics. Experience has shown that curettage of these cases is often directly harmful and may cause death within a few hours. The best treatment is thorough irrigation of the uterus; and, if possible, a partial disinfection with a strong antiseptic, such as a small amount of pure lysol swabbed over the endometrium with absorbent wool held in forceps. Such a disinfection can only be partial at the best, for the uterus bleeds freely during the process and the disinfecting fluid must be rapidly diluted and albuminised. After this the cavity should be drained by light plugging with a single strip of iodoform gauze ribbon.

Vaginal douching is useless in such cases and should be always avoided. It need hardly be said that the above treatment must be carried out with every antiseptic precaution; it is worse than useless if fresh infection is carried into the uterus from outside. Unless the surgical technique can be faithfully carried out the local treatment had better be omitted.

#### General Treatment.

General treatment is the next consideration. If the bacteriological examination reveals a streptococcus infection antistreptococcic serum is of the greatest value. The polyvalent sera manufactured from many different strains of streptococci have been proved to be of much greater value than the product of a single streptococcus strain. A large dose must be given at first, say 50 c.c. Afterwards the effect may be kept up by giving smaller doses of about 10 c.c. three times a day. The serum, however, must not be given for more than three days.

In addition to this, vaccine treatment should be begun as soon as a vaccine can be prepared from the cultures made at the first examination. The vaccine must be made from the patient's own organisms. The actual dosage of the vaccine and indications for its repetition can only be gathered from estimations of the opsonic index of the patient against the infecting organisms. If the infection is mixed the vaccine must be a mixed one, made from the various organisms found. Very few cases of puerperal sepsis have hitherto been treated by the vaccine method, but from those which have been so treated and the writer's own experience the results are most encouraging, and he, therefore, feels quite justified in recommending its adoption. The difficulties of carrying out this treatment, and its costliness need not be a bar to its use. With regard to diet, the patient must be encouraged to take as much food as possible, those articles of diet being chosen which have a high nutritive value. Alcohol has long been lauded in septic infections, and there is no doubt as to its great value in puerperal sepsis. Brandy is the best means of introducing it, and the quantities given must be varied according to the patient.

